# Chronic Cognitive Impairment in AQP4+ NMOSD With Improvement in Cognition on Eculizumab: A Report of Two Cases

**DOI:** 10.3389/fneur.2022.863151

**Published:** 2022-05-13

**Authors:** Georges Saab, David G. Munoz, Dalia L. Rotstein

**Affiliations:** ^1^Department of Medicine, University of Toronto, Toronto, ON, Canada; ^2^St. Michael's Hospital, Toronto, ON, Canada

**Keywords:** Neuromyelitis Optica Spectrum Disorder (NMOSD), aquaporin-4 (AQP4), cognitive impairment, eculizumab, corpus callosum

## Abstract

Cognitive impairment may be associated with aquaporin-4 antibody positive (AQP4+) NMOSD, particularly where there is prominent cerebral, corpus callosum, or thalamic involvement. It is unclear to what extent this phenomenon may be treatable after months to years. We describe two cases of AQP4+ NMOSD with cognitive impairment persisting over more than 6 months, where cognition improved after eculizumab was initiated. In the first case, a 51-year-old woman presented with a 2-month history of cognitive decline and ataxia, and diffuse involvement of the corpus callosum on MRI. AQP4 antibody testing returned positive. Cognitive impairment persisted on therapy with mycophenolate, then rituximab. She was switched to eculizumab from rituximab 18 months after disease onset because of breakthrough optic neuritis; memory and cognitive function improved on eculizumab. In the second case, a 26-year-old woman initially presented with visual, auditory and tactile hallucinations, and impairment in activities of daily living, and was given a diagnosis of schizophrenia. Nine months later she was hospitalized for increasing confusion. MRI showed leukoencephalopathy and diffuse involvement of the corpus callosum with multiple enhancing callosal lesions. AQP4 antibody testing was positive and CSF testing for other antibodies of autoimmune encephalitis was negative. She had some improvement in cognition with high dose corticosteroids but remained significantly impaired. On follow-up, her repeat MRI showed a small new right inferomedial frontal enhancing lesion although she did not complain of any new cognitive issues, her MOCA score was 21/30, and she was started on eculizumab. Two months after eculizumab initiation she and her family reported cognitive improvement and MOCA score was 25/30. Common features of these two cases included extensive callosal involvement and an element of ongoing gadolinium enhancement on MRI. Our experience suggests the possibility that cognitive impairment may be amenable to immunotherapy in certain cases of NMOSD.

## Introduction

Neuromyelitis Optica Spectrum Disorder (NMOSD) is an inflammatory disorder of the central nervous system (CNS) that is characterized most commonly by optic neuritis and myelitis. Detection of serum antibodies to aquaporin-4 (AQP4), a water channel on the foot processes of astrocytes, distinguishes NMOSD from other CNS inflammatory diseases. Cognitive Impairment (CI) has been well established in multiple sclerosis, the most common demyelinating disease of the CNS, but it remains controversial to what extent CI is associated with NMOSD (1, 2). CI in NMOSD may be pronounced in the presence of specific brain lesions including cerebral, corpus callosum, or thalamic involvement, but also has been reported even in absence of such lesions (3). It is unclear whether CI in AQP4+ NMOSD may be treatable, particularly after months to years. Here we present two cases of patients with AQP4+ NMOSD presenting with chronic CI along with diffuse corpus callosum lesions who experienced significant improvement in cognition after starting eculizumab therapy.

## Case 1

**Figure 1:**
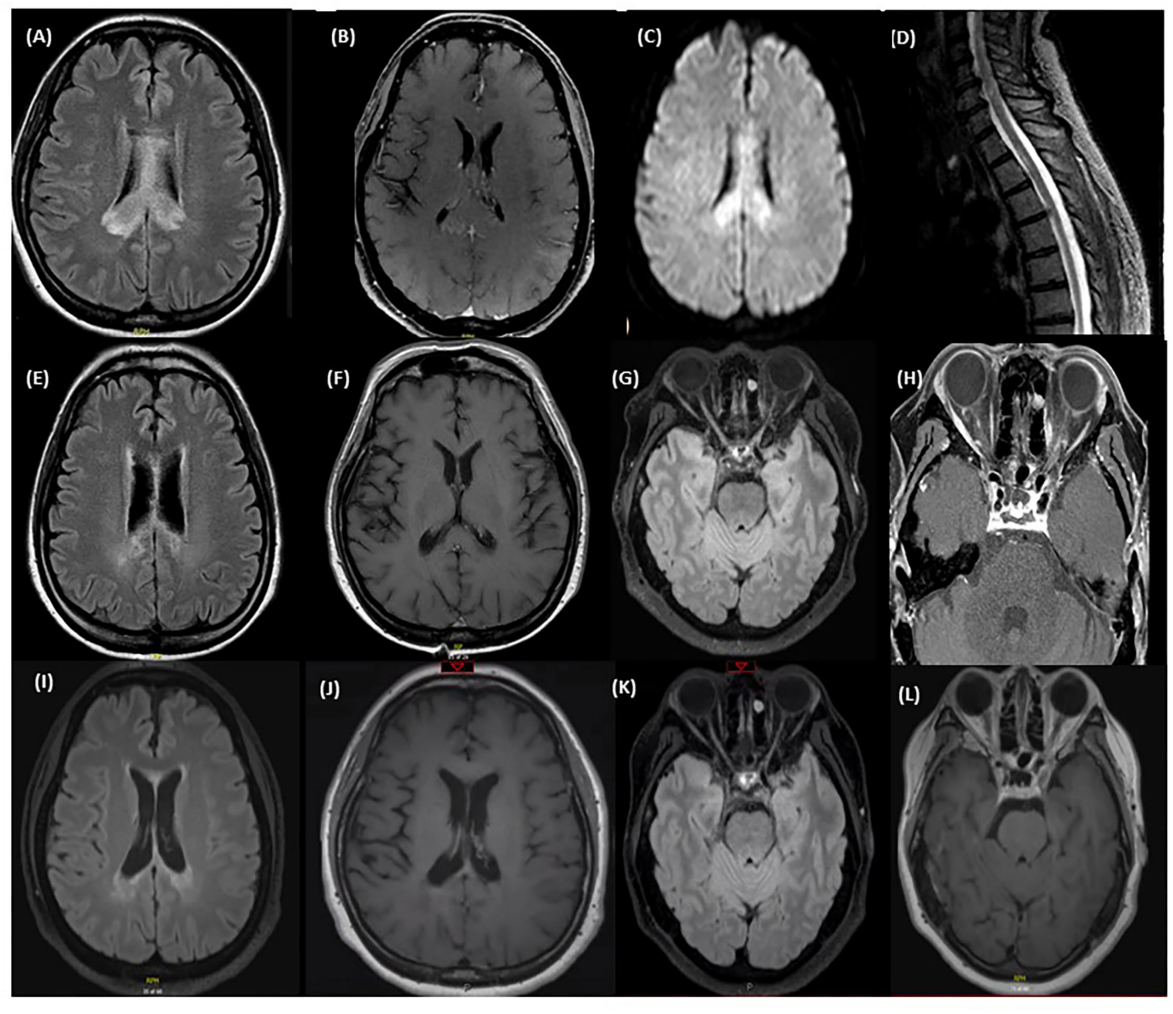
MRI at time of first relapse: **(A)** Axial T2 FLAIR sequence of the brain showing diffuse corpus callosum involvement. **(B)** Axial T1 with gadolinum showing patchy enhancement of the lesion in the right splenium of the corpus callosum. **(C)** Axial DWI sequence of the brain showing diffusion restriction. **(D)** T2 STIR sequence of the lower cervical spine and thoracic spine showing a central hyperintensity extending from the level of T1-T3. MRI after treatment of first relapse with corticosteroids: **(E)** Axial T2 FLAIR sequence of the brain showing an improvement of the corpus callosum lesions after the steroid course. **(F)** Axial T1 with gadolinium showing a resolution of the patchy enhancement after the steroid course. MRI immediately prior to eculizumab: **(G)** Axial T2 FLAIR sequence of the brain showing an increased T2 signal in the right optic nerve in the mid intraorbital course with focal atrophy of the left optic nerve. **(H)** Axial T1 with Gadolinium sequence of the orbits before starting eculizumab showing mild enhancement of both optic nerves. MRI after eculizumab: **(I)** Axial T2 FLAIR sequence of the brain showing stable deep periventricular lesions without any new lesions identified. **(J)** Axial T1 with gadolinium showing no enhancing lesions. **(K)** Axial T2 FLAIR sequence of the brain showing a decrease in the intensity of the T2 signal in the right optic nerve. **(L)** Axial T1 with gadolinium shows resolution of the previously enhancing optic nerve lesions.

A 51-year-old woman presented with a 2-month history of cognitive decline and ataxia. She complained of progressively worsening short term memory and impairment in her activities of daily living, including ability to bathe and dress herself. On neurologic exam, she was oriented to person and place, but not to time. There was evidence of truncal ataxia, bilateral intention hand tremors, and left sided dysmetria. She had an MRI of the brain that showed diffuse involvement of the corpus callosum, particularly the splenium, with diffusion restriction and patchy enhancement (Figures 1A–C), and a lesion in the right superior cerebellar peduncle. CTA of the head did not reveal any vascular abnormalities. She also had an MRI of the spinal cord, which showed a lesion in the high thoracic cord centered on the left side from T1 through T3 (Figure 1D). Cerebrospinal fluid (CSF) demonstrated 10 white blood cells/mm^3^ and protein was mildly elevated. Oligoclonal bands and culture were negative. Serum serologies for various autoimmune conditions including ANA, anti-DNA, RF, and ANCA were all negative as well. CT of the chest did not show any abnormality including hilar lymphadenopathy.

She was treated with a course of high dose IV corticosteroids followed by IVIG with mild improvement in cognition and ataxia but worsened again when steroids were tapered. Subsequently she underwent a biopsy of her brain lesions which at the time was reported to show non-specific findings of inflammation, with infiltration of macrophages and destruction of myelin and axons. She was then empirically started on mycophenolate mofetil for an inflammatory process of unknown etiology. She had gradual improvement in her ADLs and was able to resume functions including dressing and bathing herself. MRI of the brain showed an improvement in the lesions with a resolution of the enhancement (Figures 1E,F).

**Figure 2:**
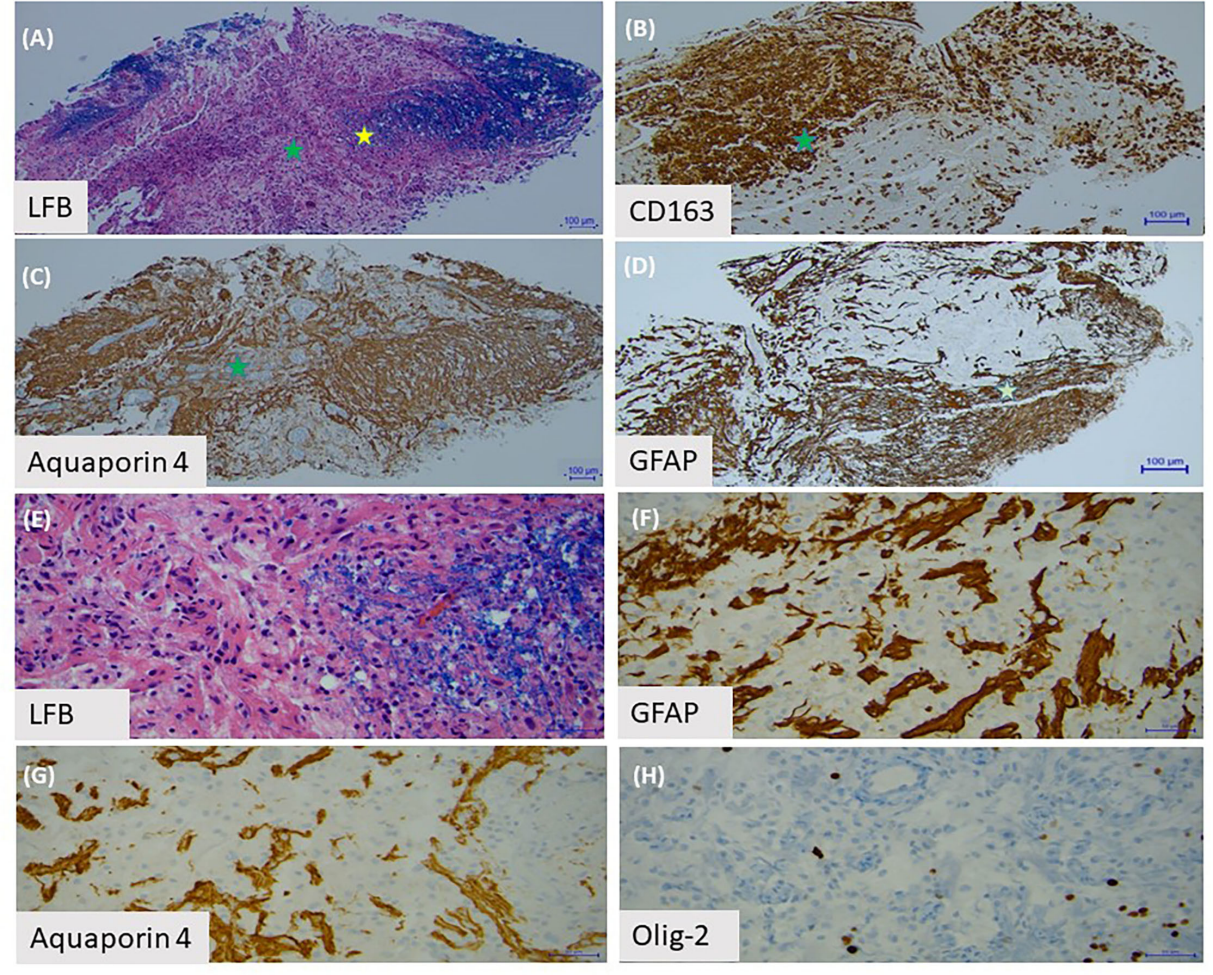
Brain biopsy with **(A)** Luxol Fast Blue staining of myelin shows a sharply demarcated area of myelin loss. **(B)** This area is infiltrated by lipid-laden macrophages, labeled by CD163. **(C)** The area with the green star demonstrates partial loss of aquaporin-4 astrocytes. **(D)** There is also depletion of GFAP positive astrocytes in this area. **(E)** Under high power (yellow star area on first slide), we observed the edge of the demyelinated lesion. **(F)** There are sparse GFAP positive astrocytes and **(G)** aquaporin-4 positive astrocytes present around blood vessels. **(H)** Olig-2 stain shows loss of oligodendrocytes.

One year after her initial presentation she developed blurry vision in her right eye, followed by complete vision loss in her left eye several days later. Exam showed no light perception in the left eye and she was only able to count fingers in her right eye. MRI of the brain and orbits revealed enhancement of both optic nerves in their orbital portions. She was treated with a course of high dose IV steroids followed by IVIG with an improvement in her right eye vision. A serum cell-based AQP4 antibody test was sent and returned positive, confirming a diagnosis of AQP4+ NMOSD. Her former biopsy slides were obtained and reviewed again; there was extensive loss of AQP4+ and GFAP+ astrocytes supporting the diagnosis of NMOSD (Figures 2A–D). In addition, sparse AQP4+ and GFAP+ astrocytes were observed in clusters around blood vessels, perhaps suggesting an early repair phenomenon (Figures 2E–H).

The patient's maintenance immunotherapy was switched to rituximab because of the breakthrough relapse. After 2 cycles of rituximab she was found to have worsening vision in her right eye from 20/80 to 20/160 along with new enhancement along the intraorbital portions of the optic nerves consistent with a bilateral optic neuritis (Figures 1G,H). She had remained cognitively impaired on rituximab with short-term memory issues and inability to take care of independent activities of daily living such as meal preparation and banking. She was switched to eculizumab. Repeat MRI brain showed no new lesion and resolution of the enhancement in the optic nerves (Figures 1I–L). The patient reported a progressive improvement in her memory and cognition, and ability to newly take on household organization, journal writing, and some banking. On follow-up 1 year after starting eculizumab, her MOCA score was 26/30.

## Case 2

A 26-year-old woman was initially seen by psychiatry 1 year prior to neurologic consultation because of paranoid visual, auditory, and tactile hallucinations. She was diagnosed with schizophrenia and started on multiple antipsychotics including aripriprazole, ziprasidone, and risperidone, but found to have an increase in her compulsions and severe nausea and vomiting. She was switched to risperidone with resolution of the nausea and vomiting. Six months later she became more acutely confused and complained of severe headaches. She was admitted to hospital and neurology was consulted. On exam she was drowsy, had stuttering and hesitant speech and evidence of psychomotor retardation. However, she was alert to person, place and time. Fundoscopy was unremarkable. She had some mild give-way weakness in all limbs without a clear pyramidal distribution.

**Figure 3:**
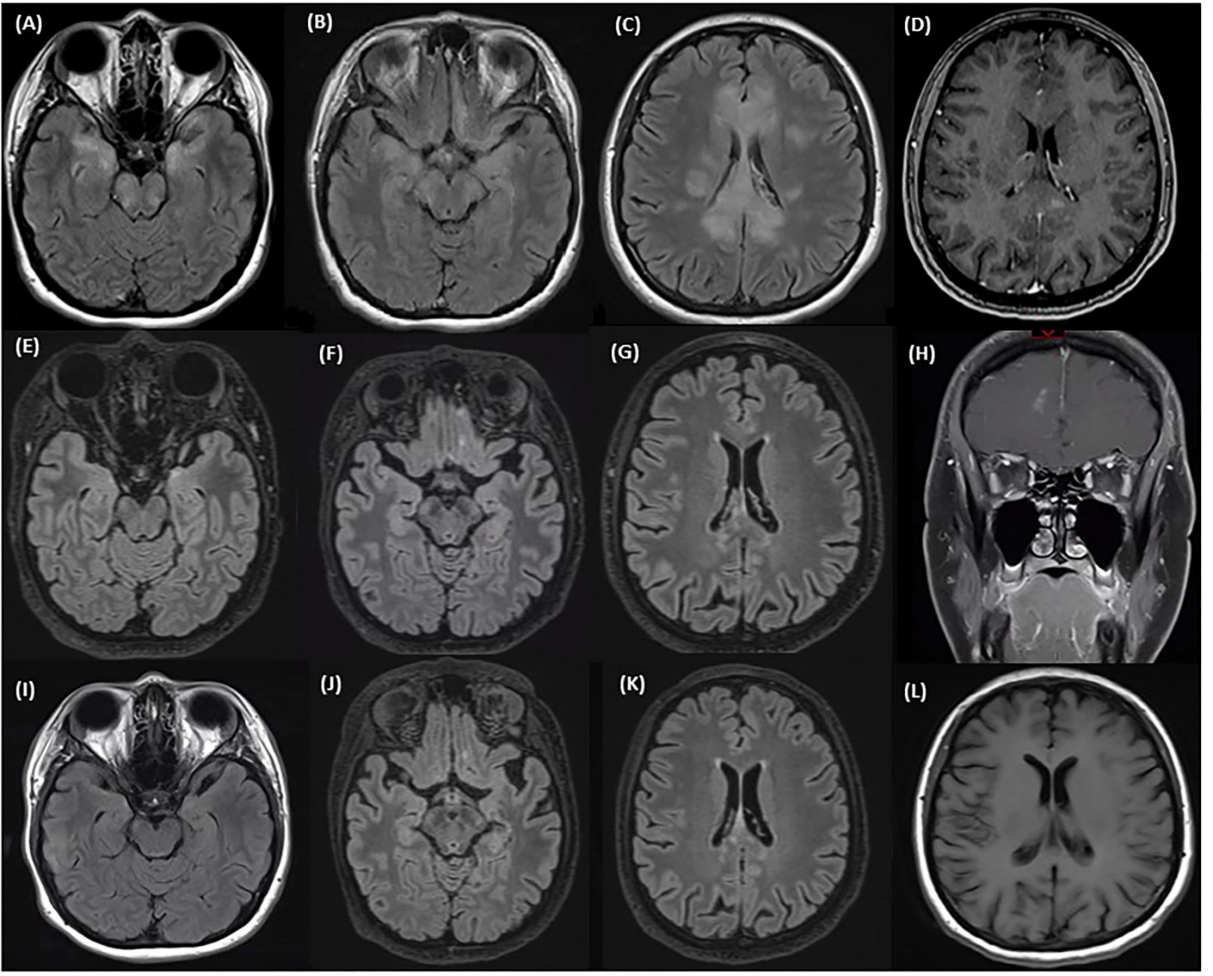
MRI at time of first relapse: **(A–C)** Axial T2 FLAIR sequence of the brain showing involvement of the corpus callosum, bilateral mesial temporal lobes, bilateral inferior frontal lobes, and bilateral pons and midbrain. **(D)** Axial sequence of the brain with gadolinium showing discrete enhancing lesions in the corpus callosum. MRI after treatment of first relapse with corticosteroids and prior to eculizumab: **(E–G)** Axial T2 FLAIR sequences of the brain showing an improvement in the mesial temporal lobe and corpus callosum lesions after a course of steroids. **(H)** Coronal T1 sequences with gadolinium showing a new small right inferomedial frontal lesion with patchy enhancement. MRI after eculizumab: **(I–K)** Axial T2 FLAIR sequences of the brain showing stable inferior frontal and temporal lobe and corpus callosum lesions after starting eculizumab. **(L)** Axial T1 sequences with gadolinium showing no enhancement.

MRI of the brain revealed T2 hyperintensities in the left frontal lobe, left insula, bilateral basal ganglia, corpus callosum, bilateral mesial temporal lobes, bilateral inferior frontal lobes, and bilateral pons and midbrain (Figures 3A–C). Lesions were fairly symmetric although there was more extensive involvement of the left frontal lobe. There were discrete enhancing lesions visualized in several places in the corpus callosum (Figure 3D). Her CSF revealed 34 white blood cells/mm^3^, 50% neutrophils, protein 1.16 g/L, and glucose 2.4 mmol/L. ANA was positive at 1:80 and SSA was positive as well. A CSF autoimmune encephalitis panel including NMDA receptor antibody testing and CSF cytology were negative. Serum syphilis, HIV, Lyme disease, West Nile virus, Influenza, and COVID-19 serologies, and MOG antibody were all negative. Her serum AQP4 cell-based antibody test returned strongly positive. She was initially treated with antivirals and antibiotics for a presumed encephalitis, then later with a course of high dose steroids with improvement in alertness and concentration, although she remained significantly cognitively impaired and unable to perform independent activities of daily living. She was not treated with an oral prednisone taper because of her psychiatric history.

On follow-up, repeat MRI showed a small, 7 mm, new right inferomedial frontal lesion with patchy enhancement, although she did not complain of any new cognitive issues (Figures 3E–H). Her MOCA score was 21/30. Eculizumab therapy was initiated. Two months after eculizumab initiation she and her family reported cognitive improvement and her MOCA score was 25/30. However, due to persistent hallucinations, she remained unable to work. MRI of the brain showed stable lesions with a resolution of the enhancement (Figures 3I–L).

## Discussion

Cognitive and psychiatric presentations of NMOSD are relatively rare but may be underrecognized. In one Korean series, 10% of patients with NMOSD presented with an encephalopathic syndrome and diagnosis was often delayed until subsequent presentation with optic neuritis (4). Psychiatric symptoms reported in association with NMOSD have included hallucinations and confusion as were observed in our second patient (5). Catatonia has also been reported (6). Cognitive and psychiatric presentations of NMOSD may lead to permanent disability due to delayed recognition and misdiagnosis. Common localization for cognitive and psychiatric relapses in AQP4+ NMOSD have included lesions in the cerebral hemispheres (4), diencephalon (5), and corpus callosum (7), and both of our cases demonstrated prominent involvement of the latter. There have been reported cases of autoimmune encephalitis, in particular NMDA receptor antibody positive encephalitis, co-existing with AQP4+ NMOSD (8, 9). This was considered in our second case because of the patient's psychiatric symptoms and confusion, but autoimmune encephalitis panel was negative. To summarize, NMOSD should be considered in the differential diagnosis of subacute onset of cognitive and psychiatric symptoms where imaging is suggestive of an inflammatory cause, and serologic testing for the AQP4 antibody testing should be performed so as not to delay diagnosis.

Corpus callosal lesions are relatively common in NMOSD; recent cohorts suggest a prevalence of up to 20% (10, 11). Such lesions have a distinct appearance from corpus callosal lesions in multiple sclerosis (MS), as they are often diffuse, cystic, oriented along the axis of the corpus callosum, and may demonstrate heterogenous signal intensity and blurred margins (12, 13). Enhancement patterns are frequently heterogenous as well. “Bridge arch” lesions of the corpus callosum have been reported, particularly in the splenium, and this appearance can sometimes be confused for a neoplastic process such as lymphoma or “butterfly” glioma (12, 13). Differential diagnosis of corpus callosum syndromes includes inflammatory causes such as MOGAD, Susac syndrome, and neurosarcoidosis in addition to NMOSD; ischemia; hereditary leukoencephalopathy; Marchiafava-Bignami disease; as well as neoplastic causes. The corpus callosum lesions seen in both our patients were more suggestive of NMOSD than MS given diffuse involvement of the corpus callosum and heterogeneous blurred margins.

Corpus callosal damage has been associated with cognitive impairment in multiple diseases including MS and Alzheimer's disease (14, 15), and was likely a major contributor to cognitive impairment in our two patients. However, cognitive deficits may occur in AQP4+ NMOSD even in the absence of a history of cognitive relapse and/or brain lesions. Recent observational studies suggest a wide range of 30–70% of NMOSD participants with CI (2, 3, 16, 17). In a cross-sectional study, no association was observed between frequency of brain lesions or overall brain T2 lesion load between cognitively preserved and cognitively impaired patients with NMOSD (18). A pathological study of NMOSD brains found evidence of diffuse cortical neuronal loss despite absence of cortical demyelinating lesions (1). The authors suggested that loss of astrocytes may be implicated in release of excitotoxic and neurotoxic factors affecting cortical neurons (1). CI in NMOSD may involve multiple cognitive domains including memory, attention, and speed of information processing (3). Neural correlates of the cognitive impairment in NMOSD have been attributed to focal reductions in white matter volume and integrity in some studies (19, 20). Other studies have suggested that focal hippocampal or thalamic volume loss may be critical to CI in NMOSD (18, 21). Further study of CI in AQP4+ NMOSD is necessary to clarify prevalence, localization, evolution, and pathologic basis of this process.

Moreover, it is unclear whether chronic CI in AQP4+ NMOSD may be amenable to immunotherapy, and to what extent such a response may depend on the duration of CI and associated MRI features including localization of lesions and the presence of gadolinium enhancing lesions. Acute cognitive and psychiatric relapses of NMOSD have been reported to improve with immunotherapy including high dose corticosteroids and IVIG (5). To our knowledge, these are the first reported cases of chronic CI in AQP4+ NMOSD with improvement on escalation of immunotherapy. The response to eculizumab in our cases supports the importance of the complement pathway in the underlying pathophysiology of AQP4+ NMOSD (22). Common features to our two cases include diffuse involvement of the corpus callosum and on-going gadolinium enhancement on MRI. Both patients demonstrated significant improvement in cognitive function after starting eculizumab, and our first patient reported an improvement in her quality of life with ability to participate in activities like meal preparation, journal writing, and banking which she had not performed for the preceding 2 years since onset of her NMOSD. Neither patient returned to her cognitive baseline however, and neither was able to return to work.

It is possible that the cognitive improvement observed in our two cases was due to suboptimal treatment of active inflammation given ongoing gadolinium enhancement on MRI. On the other hand, there was only minor change on MRIs compared before and after institution of eculizumab, and, for Case 1, the enhancement was in the optic nerves, not in a region associated with cognition. A recent histopathologic study of autopsied AQP4+ NMOSD brain specimens showed that while complement deposition is most marked at the time of relapse, there was deposition of a complement degradation product, C3d, in fibrous gliosis, a chronic stage of NMOSD lesions (23). This finding may support the possibility of chronic pathology as a therapeutic target in AQP4+ NMOSD, although this needs to be further explored. The presence of subclinical disease activity in AQP4+ NMOSD—and its potential contribution to chronic NMOSD symptoms like fatigue and CI—remains controversial. Biomarkers like serum glial fibrillary acid protein (sGFAP) levels are under investigation, as elevated levels may predict the risk of future attacks as was observed in participants in the N-MOmentum trial (24). Symptom burden was increased in N-MOmentum participants with higher sGFAP levels as well even in the absence of an adjudicated attack.

Our two cases suggest that eculizumab should be considered as a therapy in AQP4+ NMOSD patients with CI, even when CI has been present for up to 1–2 years, and particularly if there is on-going gadolinium enhancement on MRI. Taking a broader perspective, and given the prevalence of cognitive deficits in NMOSD, we wonder if regular cognitive assessments may allow for better evaluation of disease severity, functional impact, response to therapy, and more timely escalation in therapy, where chronic impairment or decline is observed. The possibility of cognitive improvement in response to certain therapies such as eculizumab in AQP4+ NMOSD warrants further consideration. This question could potentially be answered by a multi-center, observational study with formal cognitive assessments and imaging before and after initiating specific long-term therapies.

## Data Availability Statement

The original contributions presented in the study are included in the article/supplementary material, further inquiries can be directed to the corresponding authors.

## Ethics Statement

Written informed consent was obtained from the individual(s) for the publication of any potentially identifiable images or data included in this article.

## Author Contributions

GS drafted the manuscript. DM analyzed the biopsy specimen and reviewed the manuscript. DR drafted the manuscript and reviewed it. All authors contributed to the article and approved the submitted version.

## Conflict of Interest

DR has received research support from the MS Society of Canada, Consortium of Multiple Sclerosis Centers and Roche. She has received speaker or consultant fees from Alexion, Biogen, EMD Serono, Novartis, Roche, and Sanofi Aventis. The remaining authors declare that the research was conducted in the absence of any commercial or financial relationships that could be construed as a potential conflict of interest.

## Publisher's Note

All claims expressed in this article are solely those of the authors and do not necessarily represent those of their affiliated organizations, or those of the publisher, the editors and the reviewers. Any product that may be evaluated in this article, or claim that may be made by its manufacturer, is not guaranteed or endorsed by the publisher.

## References

[1] Saji E Arakawa M Yanagawa K Toyoshima Y Yokoseki A Okamoto K. Cognitive impairment and cortical degeneration in neuromyelitis optica. Ann Neurol. (2013) 73:65–76. 10.1002/ana.23721 23378324

[2] Kim S-H Kwak K Jeong IH Hyun J-W Jo H-J Joung A. Cognitive impairment differs between neuromyelitis optica spectrum disorder and multiple sclerosis. Mult Scler. (2016) 22:1850–8. 10.1177/1352458516636246 26920380

[3] Oertel FC Schließeit J Brandt AU Paul F. Cognitive impairment in neuromyelitis optica spectrum disorders: a review of clinical and neuroradiological features. Front Neurol. (2019) 10:608. 10.3389/fneur.2019.00608 31258505 PMC6587817

[4] Kim W Kim SH Lee SH Li XF Kim HJ. Brain abnormalities as an initial manifestation of neuromyelitis optica spectrum disorder. Mult Scler. (2011) 17:1107–12. 10.1177/1352458511404917 21543554

[5] Tang H Wang L Zhou H Hao X. Psychiatric symptoms as initial manifestation in neuromyelitis optica spectrum disorder without cortical lesions: a report of two cases. J Neuroimmunol. (2021) 359:577693. 10.1016/j.jneuroim.2021.577693 34403863

[6] Alam A Patel R Locicero B Rivera N. Neuromyelitis optica presenting with psychiatric symptoms and catatonia: a case report. Gen Hosp Psychiatry. (2015) 37:274.e271–2. 10.1016/j.genhosppsych.2015.02.007 25835509

[7] Camera V Messina S Elhadd KT Sanpera-Iglesias J Mariano R Hacohen Y. Early predictors of disability of paediatric-onset AQP4-IgG-seropositive neuromyelitis optica spectrum disorders. J Neurol Neurosurg Psychiatry. (2022) 93:101–11. 10.1136/jnnp-2021-327206 34583946

[8] Sinani AA Maawali SA Alshekaili J Kindi MA Ramadhani KA Khabouri JA. Overlapping demyelinating syndrome (Neuromyelitis optica spectrum disorders NMOSD with anti-NMDA receptor encephalitis); A case report. Multiple Sclerosis Relat Disord. (2020) 42:102153. 10.1016/j.msard.2020.102153 32413838

[9] Tao S Zhang Y Ye H Guo D. AQP4-IgG-seropositive neuromyelitis optica spectrum disorder (NMOSD) coexisting with anti-N-methyl-D-aspartate receptor (NMDAR) encephalitis: a case report and literature review. Multiple Sclerosis Relat Disord. (2019) 35:185–92. 10.1016/j.msard.2019.07.008 31398657

[10] Makino T Ito S Mori M Yonezu T Ogawa Y Kuwabara S. Diffuse and heterogeneous T2-hyperintense lesions in the splenium are characteristic of neuromyelitis optica. Mult Scler. (2013) 19:308–15. 10.1177/1352458512454772 22809881

[11] Carnero Contentti E Daccach Marques V Soto de Castillo I Tkachuk V Antunes Barreira A Armas E. Frequency of brain MRI abnormalities in neuromyelitis optica spectrum disorder at presentation: a cohort of Latin American patients. Mult Scler Relat Disord. (2018) 19:73–8. 10.1016/j.msard.2017.11.004 29156226

[12] Clarke L Arnett S Bukhari W Khalilidehkordi E Jimenez Sanchez S O'Gorman C. MRI patterns distinguish AQP4 antibody positive neuromyelitis optica spectrum disorder from multiple sclerosis. Front Neurol. (2021) 12:722237. 10.3389/fneur.2021.722237 34566866 PMC8458658

[13] Nakamura M Misu T Fujihara K Miyazawa I Nakashima I Takahashi T. Occurrence of acute large and edematous callosal lesions in neuromyelitis optica. Mult Scler. (2009) 15:695–700. 10.1177/1352458509103301 19435750

[14] Granberg T Martola J Bergendal G Shams S Damangir S Aspelin P. Corpus callosum atrophy is strongly associated with cognitive impairment in multiple sclerosis: results of a 17-year longitudinal study. Mult Scler. (2015) 21:1151–8. 10.1177/1352458514560928 25480866

[15] Hamphel H Teipel SJ Alexander GE Horwitz B Teichberg D Schapiro MB. Corpus callosum atrophy is a possible indicator of region- and cell type-specific neuronal degeneration in Alzheimer disease: a magnetic resonance imaging analysis. Arch Neurol. (1998) 55:193–8. 10.1001/archneur.55.2.193 9482361

[16] Moore P Methley A Pollard C Mutch K Hamid S Elsone L. Cognitive and psychiatric comorbidities in neuromyelitis optica. J Neurol Sci. (2016) 360:4–9. 10.1016/j.jns.2015.11.031 26723962

[17] Meng H Xu J Pan C Cheng J Hu Y Hong Y. Cognitive dysfunction in adult patients with neuromyelitis optica: a systematic review and metaanalysis. J Neurol. (2017) 264:1549–58. 10.1007/s00415-016-8345-3 27909800

[18] Liu Y Fu Y Schoonheim MM Zhang N Fan M Su L. Structural MRI substrates of cognitive impairment in neuromyelitis optica. Neurology. (2015) 85:1491–9. 10.1212/WNL.0000000000002067 26423432

[19] He D Wu Q Chen X Zhao D Gong Q Zhou H. Cognitive impairment and whole brain diffusion in patients with neuromyelitis optica after acute relapse. Brain Cogn. (2011) 77:80–8. 10.1016/j.bandc.2011.05.007 21723024

[20] Blanc F Noblet V Jung B Rousseau F Renard F Bourre B. White matter atrophy and cognitive dysfunctions in neuromyelitis optica. PLoS ONE. (2012) 7:e33878. 10.1371/journal.pone.0033878 22509264 PMC3317931

[21] Wang Q Zhang N Yu C Li Y Fu Y Li T. Gray matter volume reduction is associated with cognitive impairment in neuromyelitis optica. Am J Neuroradiol. (2015) 36:1822–29. 10.3174/ajnr.A4403 26338914 PMC7965033

[22] Pittock SJ Berthele A Fujihara K Kim HJ Levy M Palace J. Eculizumab in aquaporin-4-positive neuromyelitis optica spectrum disorder. N Engl J Med. (2019) 381:614–25. 10.1056/NEJMoa1900866 31050279

[23] Takai Y Misu T Suzuki H Takahashi T Okada H Tanaka S. Staging of astrocytopathy and complement activation in neuromyelitis optica spectrum disorders. Brain. (2021) 144:2401–15. 10.1093/brain/awab102 33711152

[24] Aktas O Smith MA Rees WA Bennett JL She D Katz E. Serum glial fibrillary acidic protein: a neuromyelitis optica spectrum disorder biomarker. Ann Neurol. (2021) 89:895–910. 10.1002/ana.26067 33724534 PMC8252046

